# Could Short Stems THA Be a Good Bone-Saving Option Even in Obese Patients?

**DOI:** 10.3390/jcm11237114

**Published:** 2022-11-30

**Authors:** Michela Saracco, Andrea Fidanza, Stefano Necozione, Giulio Maccauro, Giandomenico Logroscino

**Affiliations:** 1 “A. Gemelli” IRCCS University Hospital Foundation, Catholic University of Sacred Heart, 00168 Rome, Italy; 2 Department of Orthopaedics, ASL Napoli 2 Nord, 80027 Naples, Italy; 3 Department Life, Health and Environmental Sciences—Mininvasive Orthopaedic Surgery, University of L’Aquila, 67100 L’Aquila, Italy; 4 Department Life, Health and Environmental Sciences—Unit of Epidemiolody, University of L’Aquila, 67100 L’Aquila, Italy

**Keywords:** osteoarthritis, obesity, mini-invasive THA, short stems, stemless hip prostheses, total hip arthroplasty, THA

## Abstract

Short femoral stems, with preservation of the femoral bone stock, are commonly used in recent years for hip replacement in younger and more active patients. Obesity is increasingly spreading even in the younger population. The aim of this case-series study is to evaluate short stems compared to traditional hip prostheses in the obese population. A total of 77 consecutive patients with a BMI greater than or equal to 30 Kg/m^2^ were enrolled in this prospective study and were divided into two groups: 49 patients have been implanted with short stems while 28 patients were implanted with traditional stems. All the patients were treated for primary osteoarthritis or avascular necrosis and all the stems were implanted by the same surgeon using a posterior approach. Clinical (Harris Hip Score—HHS, Western Ontario and McMaster Universities Osteoarthritis Index—WOMAC, visual analogue scale—VAS, 12-item Short Form Health Survey—SF-12) and radiographic outcomes were recorded. Radiological evaluations were carried out by three different blinded surgeons. A statistical analysis was performed (chi-square, t-test, Wilcoxon Rank Sum Test, 2-factor ANOVA). At a mean follow-up of 42.6 months both groups showed a marked improvement in pain and in the clinical scores between pre- and post-surgical procedures (*p* < 0.05) with no significant differences between the two groups at last follow-up (*p* > 0.05). The radiological evaluations, with high concordance correlation between the three blinded surgeons (ICC consistently >0.80), showed good positioning and osseointegration in all cases, with no significant differences in the restoration of the joint geometry and complications. No revisions were recorded during the follow-up period. In conclusion, short stems appear to be a good option for bone preservation even in obese patients, showing comparable results to traditional implants.

## 1. Introduction

Obesity is a significant and disabling disease, which affects a significant portion of the population due to poor habits and sedentary lifestyle, particularly in the most developed countries. The prevalence of obesity is increasing, and worldwide, 1.9 billion are overweight while 650 million are obese (WHO, 2016). Italy follows this trend, with about 4 million obese people and an increase of almost 30% in the last 3 decades. Previous studies estimated that 2–4% of the total health expenditure in Europe is attributed to obesity, and this is projected to double by 2050 [1]. Obesity is known to be a significant risk factor for the development of osteoarthritis (OA), often severe and of early onset, affecting the hip and knee. IL-1, TNF-α and IL-6 may cause OA indirectly by regulating the release of adiponectin and leptin from adipocytes, summarizing the relationship between obesity and inflammation [2]. Therefore, the demand for hip prostheses in young and obese patients is growing sharply. This implies executive difficulties but also doubts in choosing the most correct and safe implant, also considering the young age and the increased risk of revision due to long life expectancies. [3]

Over the last two decades, several conservative femoral prostheses have been designed for use particularly in young patients with high-activity requests. “Short stems” have been designed to be less invasive than conventional stems. Traditional femoral stems have provided successful long-term results. However, long femoral stems may have consequences related to stress shielding, thigh pain and cortical hypertrophy. Additionally, surgeons should always consider future revision, especially for young patients, because revision surgery of long stems is more invasive and require a significantly higher sacrifice of the residual bone stock. Short stems allow the preservation of more bone for future revisions and consequently are less invasive in case of revision surgery, with clear advantages and benefits for the patient and the surgeon.

The goals of short, conservative stems include saving of the trochanteric bone stock; a more physiological load in the proximal femur reduces the risk of stress shielding and avoiding the impingement of the tip of long stems with the femoral cortex with consequent thigh pain. Biomechanical studies showed that these metaphyseal-fitting stems exhibit good fixation, achieving durable bone ingrowth. Many papers on normal weight patients and short stems have been published [4,5,6,7]. Very little information is reported regarding whether short femoral stems in overweight patients offer the same reliability as in normal weight patients. [8] In particular, there are questions about the risk of increased subsidence and the fact that excessive weight can interfere with osseointegration. [9]

The purpose of this case-series is to compare two groups of obese patients, treated with traditional stems and short stems, respectively, in order to analyse the reliability and safety of the latter, not only in normal weight but even in obese patients.

## 2. Materials and Methods

We retrospectively studied a total of 77 patients who underwent primary total hip arthroplasty (THA) for end-stage hip OA. The inclusion criteria were: age between 35 and 85 years old, primary and monolateral THA and body mass index (BMI) greater than or equal to 30 Kg/m^2^. The same surgeon performed all the arthroplasties with a posterior approach with external rotator reconstruction. The indications were primary OA and avascular necrosis (AVN). The exclusion criteria were: bilateral procedures, revision surgery (aseptic loosening, periprosthetic infections or fractures), cemented stems, inflammatory diseases (i.e., rheumatoid arthritis) and/or neurological diseases (i.e., stroke, degenerative diseases). The cohort was divided into 2 groups. The study group (SS) included 48 patients who were implanted with a short metaphyseal-fitting femoral stem, belonging to III A group (subcapital osteotomy) and III B group (standard osteotomy) according to Feven and Shimmin [10], coated or not with hydroxyapatite (HA) and cementless (i.e., SMF-S&N, GTS-Zimmer Biomet, Minima-Lima, Nanos-S&N, Proxima-DePuy-J&J, Pulchra-Adler Ortho, Parva-Adler Ortho, Fitmore- Zimmer Biomet). The mean age was 63 years (43–84 years old, SD: 10.05). The mean BMI was 33.5 Kg/m^2^ (30.1–41.3, SD: 3.07) with a mean body weight of 92.3 kg (75–113 Kg, SD: 10.39). The control group (TS) included 28 patients who were implanted with traditional femoral stems belonging to type IV (traditional stems) coated or not with HA, and cementless (i.e., ABG-Stryker, Synergy-S&N, Mercurius-Adler Ortho, Hydra-Adler Ortho, Corail-DePuy-J&J). In this control group, the mean age was 67 years (50–85 years, SD: 10.03). The mean BMI was 34.7 Kg/m^2^ (30–44.5 Kg, SD: 4.68) with a mean body weight of 96 kg (67–130 kg, SD: 17.89) (Table 1).

For all the cases, baseline subjective and objective evaluations were recorded (Harris Hip Score—HHS, Western Ontario and McMaster Universities Osteoarthritis Index—WOMAC, visual analogue scale—VAS, 12-item Short Form Health Survey—SF-12 *p*). HHS is an objective and reproducible assessment method, based on the examination of two main parameters: pain and functional capacity of the hip. The secondary parameters examined are range of motion and the presence/absence of deformity. [11] WOMAC is a validated tool for measuring the symptoms and physical disability of patients suffering from hip and knee osteoarthritis. It is a self-administered questionnaire that probes clinically significant symptoms related to pain, stiffness and physical function. The questionnaire consists of 24 questions (5 on pain, 2 on stiffness and 17 on physical function) [12]. The VAS system allows the recording of the pain symptom, asking the patient to indicate a point on a straight line. Its extremes correspond to: zero pain–maximum possible pain. The SF-12 questionnaire represents a reduced version of the SF-36: it allows an estimation of physical (*p*) and mental (M) health perceived by the patient [13].

The 2 groups were compared in terms of preoperative and postoperative HHS, VAS, WOMAC and SF-12 scores.

Low-molecular-weight heparin was administered during the first 5 weeks after surgery, starting from 6 h after the procedure. In addition, 2 g of cefazolin was administered at anaesthesia induction and tranexamic acid was used intraoperatively for bleeding control.

The variables age, BMI, weight and follow-up are expressed on average (min–max) ± Standard Deviation. For each implanted device the percentage is shown in brackets.

**Figure 1:**
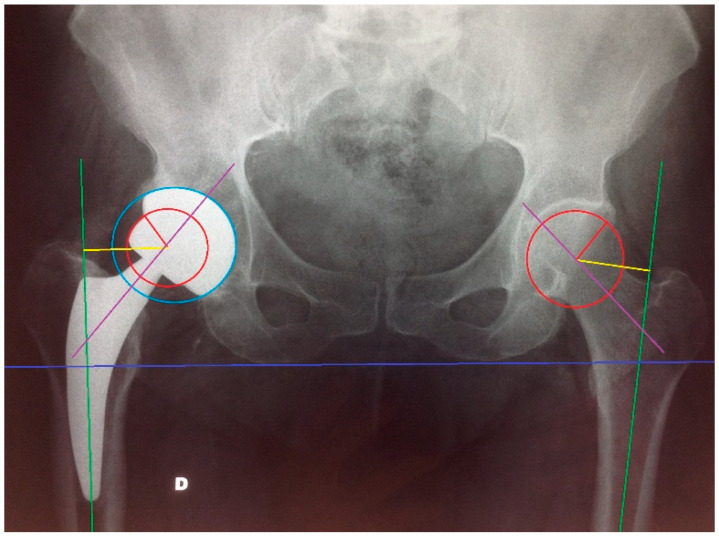
X-ray evaluation of joint geometry restoration.

For all the patients, anteroposterior (AP) pelvis radiographs were taken after the procedure and at the last follow-up visit, and all the measurements were taken by three of the authors in a blind fashion and random order using AXIOVISION 4.8.2 software (Carl Zeiss Microimaging GmbH). The post-operative pelvis X-rays were calibrated for size using the diameter of the prosthetic head or alternatively of the metal back, extracted from the operator registers. Firstly, off-set was evaluated by measuring the distance between the center of rotation of the femoral head and a line dissecting the long axis of the femur. Cervical-diaphyseal angle was evaluated as the angle between a line dissecting the long axis of the femur and a line dissecting the femur neck axis. Leg length discrepancy >1 cm was considered significant. We also evaluated the presence of subsidence on the last radiograph. Finally, we measured the cup inclination (the angle between a line dissecting the acetabular equator and the trans-ischiatic line, correctly included between 35° and 55°) and the linear polyethylene wear (Figure 1).

Stress-shielding, spot-welds, cortical hypertrophy and femoral osteolysis were graded on the radiographs at the final follow-up according to the classification of Engh, dividing the interface between the bone and the stem of the hip prosthesis into the seven zones of Gruen [14,15]. Short stem radiological outcome was assessed according to a modified Gruen zoning system, eliminating zone three and five [4,16,17]. We also evaluated the metal-back osseointegration according to the classification of Hodgkinson, dividing the interface between the bone and the metal-back into the three zones of Charnley–De Lee [18,19]. Periprosthetic heterotopic ossifications were evaluated by the classification of Brooker (from one to four) [20].

### Statistical Analysis

The distribution of the variables was tested by applying the Shapiro–Wilk test. The statistical tests performed to evaluate the initial demographic differences between the two groups were: *t-test* for normally distributed variables (age and weight), *Wilcoxon rank sum test* for non-normally distributed variables (BMI), *chi-squared test* for dichotomous variables and *Fisher’s exact test,* as appropriate (follow-up).

In order to study the trend of clinical outcomes, time per techniques interaction was calculated with *2-factor ANOVA using the repeated statement*.

Finally, to more directly interpret the differences between the two groups at baseline and at the last follow-up, *unpaired T-test* (HHS, SF-12) and *Wilcoxon rank sum test* for nonparametric data (VAS, WOMAC) were performed.

For the analyses, a statistical confidence level of 95% was selected. A *p* value < 0.05 determined significance.

## 3. Results

No differences were found between the demographic data of the two groups; they appeared homogeneous for age (*p* = 0.10), weight (*p* = 0.44) and BMI (*p* = 0.63). The mean follow-up was 38 months (3–120 months, SD: 25.98) for the SS Group and 47.3 months (12–168 months, SD: 43.15) for the TS Group (*p* = 0.77).

**Figure 2:**
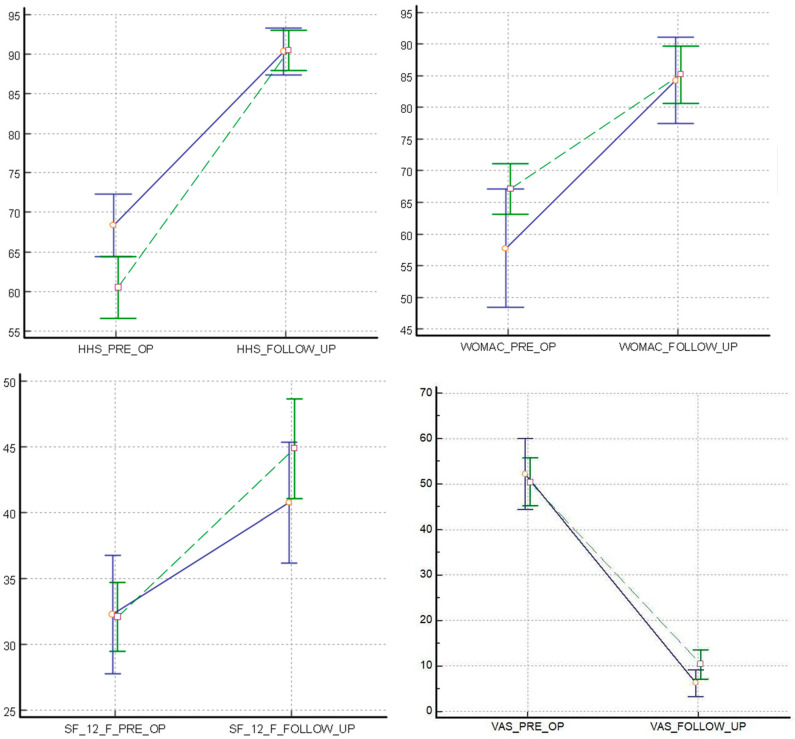
Trends in clinical scales before and after surgery in the two groups (SS—dashed green line vs TS—solid blue line). x: clinical scale evaluated; y: scores recorded. HHS: Harris Hip Score; WOMAC: Western Ontario and McMaster Universities Osteoarthritis Index; SF-12: 12-item Short Form Health Survey; VAS: Visual Analogue Scale.

All the implanted stems were well osseointegrated and positioned at the last follow-up. In both groups there was a marked improvement in all the parameters compared to the preoperative conditions. The difference between pre- and post-surgery was statistically significant for all the clinical scores evaluated (*2-factor ANOVA using the repeated statement)*, and there were no significant differences between the two groups at last follow-up (*unpaired T-test* and *Wilcoxon rank sum test).* The statistical values of each analysed variable are shown below and represented in the graphs of Figure 2.

Pain was significantly reduced in both groups, VAS (SS vs. TS) decreased from 49.8 to 9.2 and from 51.3 to 5.4 (*p* <0.001), with no significant differences between the two groups at last follow-up (*p* = 0.099).

HHS SS increased from 60.5 to 90.4—TS from 68.4 to 90.3 (*p* <0.001) without statistical differences at the last control (*p* = 0.94). WOMAC also increased after the surgery in both groups: SS from 67.1 to 85.2—TS from 57.7 to 84.3 (*p* <0.001) with no differences at last follow-up (*p* = 0.816). SF-12 *p* increased in group SS from 32.1 to 44.9 SS and from 32.3 to 40.8 in group ST (*p* <0.001), with a final *p* = 0.16.

The radiological evaluations showed high concordance correlation between the three blinded surgeons (intraclass correlation coefficient (ICC) consistently >0.80) and no significant difference between the two groups in the ability to restore proper articular geometry. The average value of the C-D angle of the side undergoing arthroplasty with long stem was 136.4°, while that of the contralateral was 133.1° with a statistically insignificant difference (*p*: 0.41); the same can be said for short stems (SS 136.6° vs 135.1°; *p*: 0.51). As for the offset, it was on average 40.9 mm in long stems and 35.5 mm in the contralateral, with an insignificant difference given that the *p* value is equal to 0.18; on the other hand, considering the short stems, the average value was 38.3 mm, with the contralateral 36.3 mm (*p*: 0.27). No significant difference was found in hypermetria between the two groups: SS + 2.2 mm. vs ST + 2.9 mm. (*p* value: 0.85)

Even the subsidence values did not differ much and were still less than 1 cm, the value considered clinically significant. The radiographic parameter which showed a significant difference between the two groups was the cup inclination. The average inclination values were 43.9° for patients with short stems (min: 29.39°; max: 59.86 °) and 55.2° for long stems (min: 39.8°; max: 70.84°) (*p*: 0.010).

In two patients with long stems, areas of acetabular osteolysis were identified, in one patient in Chanley–De Lee zone one, while in the other was in zone three; areas of osteolysis were also found in two subjects with short stems, but in zones one and two (*p*: 0.16). Osteolysis of the femur was found in two patients in the SS group, one in zone two of Gruen and the other in zone six, and in three cases in the TS group, one in zone one and two in zone two (*p*: 0.25). A total of seven patients with long stems and 15 patients with short stems had radiographical signs of heterotopic ossifications (*p*: 0.39), without any clinical significance. Cortical hypertrophy was present in three patients with traditional stems, one in zone two of Gruen and two in zone three, and in six patients with short stems, in the area two of Gruen (*p*> 0.05). Stress-shielding was found in 11 patients with traditional stems in the zone one of Gruen, and in three of them also in zone two; a reduction in bone density was recorded in 14 patients with short stems, however in zone one of the Gruen scale, with only one patient also presenting it in zone seven.

A pedestal was observed in three long stems, and it was incomplete. No pedestals were observed in the short stems group. The spot-welds were recorded in five patients with long stems and in 15 with short stems, involving zones three and five of Gruen. Of the 77 patients studied, 10 complications were recorded: a neurological damage (one in TS), dislocations (two in SS, two in TS), infection (one in TS) and intra-operative periprosthetic fractures (one in SS, three in TS). Two fractures were metaphyseal cracks (two SS and one TS) type B1 by of the Vancouver classification and were solved by cerclages, while two other fractures occurred in the TS Group and were both A1 type that required cerclage stabilization in one case (undisplaced fracture) and ORIF (open reduction and internal fixation) with a proximal hooked plate in the other case (displaced fracture). No revision of the implant was required during the follow-up period due to implant failure, therefore, the Kaplan–Meier analysis showed a survival rate of 100% for both groups.

## 4. Discussion

The main finding of this study is that short stems, as well as traditional stems, guarantee good outcomes in THA even in overweight/obese patients. It is believed that obesity is a risk factor for osteoarthritis leading to joint replacement surgery and that body weight also affects the severity of the disease [21,22,23]. Regarding the clinical benefits of hip replacement, no significant difference between non-obese and obese people is reported in the literature since these patients seem to have great benefits regardless of their BMI [24,25,26]. In a study conducted by Jackson et al. it was found that the non-obese group had a significantly higher postoperative HHS and a greater range of motion. The researchers believe that the main reason for the difference in the range of motion is linked to the apposition of soft tissue that occurs in extreme positions, with an impact on the results of functionality and activity, but overall satisfaction after surgery was comparable or higher in in the obese patients. The greater satisfaction of obese patients is possibly explained by the fact that they start with a lower score but after surgery they will obtain a score similar to non-obese patients so that the greater difference between the preoperative and the last follow up scores leads them to a more relevant satisfaction. In addition, radiological analysis of the acetabular and femoral components did not show significant differences in the two groups [27]. Consequently, avoiding hip replacement surgery for obese patients is not justified [28]. Obese patients are often significantly younger than non-obese patients and, thanks to the good functional results of hip replacements, the indications have been extended to patients of lower age and the techniques have followed the trend for minimally invasive and bone preservation surgery [23,24]. Due to the promising biomechanics of short stems, added to the growing prevalence of obesity in the general population, it is necessary to evaluate the impact of BMI on this type of prosthesis. In our study, patients showed excellent values of HHS, VAS, WOMAC and SF-12 scales that confirm the data already present in the literature about patient satisfaction, improvement in physical activity and quality of life [29,30,31,32].

Pirard and De Lint, Todkar and Bosker et al. found no influence of the BMI on component positioning [33,34,35], while Callanan et al. and Elson et al. highlighted an association between BMI and cup mal-positioning [36,37]. In the majority of cases, only the posterior approach is used since it allows excellent exposure [38]. Few data are available on the lateral or anterolateral approach regarding cup positioning. Brodt et al. have seen how in direct lateral approach the anteversion of the cup is related to the patient’s BMI and age, assuming that the cause is the greater traction exerted by the retractors on the surrounding tissues [39]. In our study we also found a major tendency to cup malposition in the TS group. On the other hand, joint geometry was correctly restored in both groups.

Currently, there is conflicting evidence that obesity has a negative impact on the survival of the hip prostheses. In a large analysis, Culliford et al. showed that BMI has a low but statistically significant association with revision risk [40]. The reason for the increase in the rate of early failure due to aseptic loosening/osteolysis in the obese may be related to the higher mechanical stresses on the bone-implant interface and the reaction forces of the joint proportional to the body weight. Recent studies have shown that BMI has no statistically significant influence on the subsidence of uncemented short stem prostheses. Only one study has shown that body weight over 75 kg has a significant impact on subsidence and therefore on the stability of the prostheses, while there is no correlation with BMI [3,41]. To date, only few studies have focused on the relationship between BMI and functional results of short stem implants. The study by Freitag et al. analysed the relationship between BMI and functional results of short-stemmed THA, demonstrating the absence of correlation between obesity and subsidence [42]. A case-control study of the functional outcomes of Metha B-Braun prostheses demonstrated that the postoperative clinical improvement is similar in obese versus non-obese patients [8]. Hungerford et al. studied the influence of obesity on the placement and outcome of minimally invasive anterior implants and reported that there is no statistically significant difference between obese and the non-obese groups [9].

Indeed, no statistically significant difference was found in our study regarding subsidence, which is less than 2 mm in both groups. This evidence further supports that the stability of implants is not influenced by obesity. Furthermore, both groups showed few cases of osteolysis and no significant differences were found on leg length discrepancy. No statistically significant differences were found on osseointegration, with excellent results in both groups. Regarding the incidence of heterotopic ossifications, Andrew et al. noted that there is no statistically significant difference between the obese and the non-obese [43]. We found heterotopic ossifications in both groups without any significant functional limitation or clinical relevance.

Although the literature on the post-operative risk of thromboembolic events documents an increasing risk in obesity, this is not consistently replicated in the orthopaedic literature [44,45,46]. Similarly, it has been empirically shown that the risk of dislocation increases with extreme excursions of the joint angle and fat tissues around the hip [47]. Paradoxically, however, obese patients have structural and functional limitations that tend to reduce the excursion of the hip during walking and daily life activities. In our study, few patients reported this complication.

The association between obesity and wound infections is reported in the current literature, including orthopaedic procedures [48,49]. In a recent large-scale study, an association between BMI and infection after THA was initially reported. However, when the influence of coexisting diseases such as diabetes mellitus was no longer taken into account, obesity was no longer an independent risk factor for infection [50]. Dowsey and Choong demonstrated the existence of a significantly higher incidence of acute periprosthetic infection after primary hip arthroplasty in obese and super-obese patients compared to non-obese patients [51,52,53]. There is also an increased risk of wound dehiscence due to increased surface tension, as well as hematoma formation correlated with prolonged wound drainage [54,55,56]. In our study, previous or active periprosthetic infection was a criterion for the exclusion of patients and we recorded only one case of post-surgical superficial wound infection. Since obesity is a known risk factor for wound complications and regardless of the surgical approach, it is essential to identify the best surgical approach in patients with high BMI to limit the risks related to surgery [57,58]. Previous studies suggested that the risk for obese patients is relatively higher following a direct anterior approach when compared to a posterolateral approach (particularly BMI ≥40 kg/m^2^) [59]. Consequently, in obese patients, the choice of a posterolateral approach is therefore advisable, and for this reason it was chosen for all the patients in our study.

## 5. Conclusions

The results of this study demonstrate that short stems in total hip arthroplasty are a valid option in obese patients in whom, until now, traditional long-stem prostheses have been commonly implanted. Short stems were showed to be able to withstand overload, allowing excellent osseointegration and implant stability. Considering that obese patients have an early onset of osteoarthritis and undergo earlier to THA, and that they are more greatly subjected to the risk of failure or complications, short stem implants preserving bone may represent an advantage in case of revision surgery and in long life-expectancy patients. Due to the limitations of our study, no definitive judgment can be made; however, this study may represent the basis for future studies with a larger sample and longer follow-up to provide more solid statistical evidence.

## Figures and Tables

**Table 1:** Demographics of our sample and implanted devices. The case study group includes 48 patients undergoing THA with short stems. The control group includes 28 patients who underwent THA with standard traditional stems.

	Short Stems (SS)	Traditional Stems (TS)
AGE, years	63 (43–84) ± 10.05	67 (50–88) ± 10.03
BMI, Kg/m^2^	33.5 (30.1–41.3) ± 3.07	34.7 (29.8–44.5) ± 4.68
WEIGHT, Kg	92.3 (75–113) ± 10.39	95.9 (67–130) ± 17.89
FOLLOW-UP, months	38 (3–120) ± 25.98	47.3 (12–168) ± 43.15
PARVA, Adler	20 (40%)	-
PROXIMA, DePuy-J&J	7 (16%)	-
MINIMA, Lima	7 (16%)	-
FITMORE, Zimmer Biomet	4 (10%)	-
PULCHRA, Adler	3 (6%)	-
SMF, S&N	3 (6%)	-
GTS, Zimmer Biomet	2 (3%)	-
NANOS, S&N	2 (3%)	-
ABG, Stryker	-	9 (59%)
SYNERGY, S&N	-	6 (17%)
MERCURIUS, Adler	-	5 (12%)
HYDRA, Adler	-	4 (6%)
CORAIL, DePuy-J&J	-	4 (6%)
